# Effect of Abdominal Adiposity on the Impact of Plantar Force in the Foot Support of Obese and Overweight Schoolchildren

**DOI:** 10.3390/children12111553

**Published:** 2025-11-17

**Authors:** Ana Paula Ribeiro, Daniel Borges Pereira, Gabrielle Fontura Berger, Kemely Muraiber Ismail, Maitê Duarte Morais, Mayara Slaiman Fares Martins

**Affiliations:** 1 Biomechanics and Musculoskeletal Rehabilitation Laboratory, Health Science Post-Graduate Department, Medicine School, University Santo Amaro, São Paulo 04829-300, SP, Brazil; daniel.borges@estudante.unisa.br (D.B.P.); gfontour@estudante.unisa.br (G.F.B.); kemely@estudante.unisa.br (K.M.I.); maite1w@estudante.unisa.br (M.D.M.); mslaiman@estudante.unisa.br (M.S.F.M.); 2 Physical Therapy Department, School of Medicine, University of São Paulo, São Paulo 01246-903, SP, Brazil

**Keywords:** children, obesity, overweight, foot, force, posture

## Abstract

**Highlights:**

**What are the main findings?**

• Abdominal adiposity, measured via ultrasound, was a strong predictor of increased foot pronation (on both right and left sides);
• A high-to-moderate association was observed between abdominal fat thickness and pronated foot support.

**What are the implications of the main findings?**

• Abdominal adiposity contributes directly to postural and orthopedic changes in children, specifically pronated foot support.
• Changes in foot posture observed in overweight and obese children may predispose them to pain and orthopedic complications during growth, highlighting the critical need for early assessment and intervention.

**Abstract:**

**Background**: Childhood obesity is a growing global concern associated with early-onset orthopedic complications that may persist into adulthood. Among the anthropometric indicators, abdominal adiposity plays a key role in assessing health risks during pediatric evaluations. However, the relationship between abdominal fat distribution and biomechanical alterations, such as changes in posture and foot support, remains poorly understood. Ultrasonography (US) is a validated, noninvasive imaging method capable of distinguishing preperitoneal and intraperitoneal fat in children. Despite its diagnostic advantages, no study to date has directly examined ultrasound-measured abdominal adiposity-predicted pronated foot posture in children. **Objective**: We aimed to verify the impact of abdominal adiposity on foot support and its association with obese, overweight, and eutrophic schoolchildren. **Methods**: This is a cross-sectional study. Sixty-five pupils (aged 6–9 years) from a public school in São Paulo, Brazil, were divided into three groups according to nutritional status: obese (n = 25), overweight (n = 20), and eutrophic (n = 20). Anthropometric measurements and foot posture, assessed using the Foot Posture Index (FPI), were collected during the initial evaluation. Abdominal adiposity was determined by ultrasonography, measuring subcutaneous, preperitoneal, and intraperitoneal fat thickness. **Statistical Analysis**: Analyses compared group differences and relations between abdominal fat and foot posture, with significance set at *p* < 0.05. **Results**: Obese and overweight schoolchildren showed pronated foot posture when compared to eutrophic children, on both sides of the feet. Abdominal adiposity was a good predictor of a more pronated footrest for the right and left feet, showing a high-to-moderate association. **Conclusions**: Ultrasound-measured abdominal adiposity was identified as a significant predictor of pronated foot posture in schoolchildren. These findings highlight the importance of monitoring abdominal fat accumulation during pediatric evaluations, as excessive adiposity may increase the risk of musculoskeletal dysfunctions and pain in the lower limbs. Early detection of these alterations may help prevent postural and musculoskeletal disorders in overweight and obese children.

## 1. Introduction

Pediatric obesity, characterized by excess body weight or adiposity, is one of the most serious public health challenges of the 21st century, affecting populations across Asia, Latin America, and the United States [1,2]. It persists in both developing and developed countries, with significant economic and social consequences [2,3]. Since the 1970s, the prevalence of overweight and obesity among children, adolescents, and adults has increased worldwide [4], currently affecting around 340 million individuals aged 5 to 19 years—approximately 18% globally and 19% among males [1,4]. This alarming trend is attributed mainly to poor nutrition and physical inactivity among schoolchildren, contributing to sedentary behavior and excess weight gain [5]. The COVID-19 pandemic further intensified these risks by reducing children’s mobility and physical activity due to school closures and social isolation, while also worsening dietary habits and emotional eating [5,6]. Consequently, the world faced a “collision of two pandemics”: COVID-19 and obesity [7,8].

Given this scenario, health professionals and policymakers have expressed growing concern about developing effective strategies for the prevention and management of childhood obesity [3,4,8]. The condition has been linked to multiple comorbidities, including cardiovascular diseases such as hypertension and dyslipidemia, pulmonary disorders like sleep apnea, and gastrointestinal issues such as fatty liver disease [9]. Furthermore, obese and overweight children show higher medication use, greater demand for outpatient and emergency services, and increased healthcare costs [10,11,12,13]. In Brazil alone, the Unified Health System (SUS) spent $269.6 million on obesity-related care in 2011, with nearly 24% attributed to morbid obesity [12,13].

In addition to metabolic complications, obesity exerts mechanical overload on the musculoskeletal system, promoting postural alterations, reduced motor coordination, and pain in the lower limbs [12,14,15,16]. It is also associated with orthopedic conditions such as femoral head epiphysitis, fractures, ankle sprains, and joint inflammation, especially in the knee and ankle [17,18,19,20], as well as peripheral nerve compression due to excessive muscle tension [21]. These complications increase joint load and muscular demand along the lower kinetic chain, compromising both static and dynamic activities [21,22]. Research has shown that obese children exhibit reduced thoracic flexion, greater hip joint loading, and altered trunk posture due to abdominal protrusion and anterior displacement of the body’s center of gravity [14,16,22,23,24]. The most frequent postural deviations include anterior head projection, pelvic anteversion, cervical and lumbar hyperlordosis, dorsal kyphosis, and valgus alignment of the knees and ankles with worse flexibility [1,10,11,12,14,15,22,23]. Pelvic anteversion may lead to femoral medial rotation, increased knee valgus, and plantar arch collapse, resulting in excessive foot pronation and rearfoot valgus [1,22,23,24].

To maintain balance, obese children often adapt their gait and postural control. Aleixo et al. (2012) [24] reported reduced dynamic balance and lower limb dissociation in schoolchildren with obesity, which led to increased plantar pressure during walking [25,26]. Several studies have examined foot posture in overweight and obese children, given that excess body mass and reduced coordination may overload the feet, causing pain and discomfort in specific regions [25,26,27]. During normal development, the medial plantar arch matures around 6–7 years of age [27], but obesity can interfere with this process. Most studies have observed more pronated or flat feet among obese and overweight children [27], though others found no significant differences compared to their eutrophic peers [28,29,30,31]. For instance, Jiménez-Ormeño et al. (2013) [28] reported that although overweight and obese children had larger and wider feet, there was no correlation between BMI and the plantar arch index: a result consistent with Carvalho et al. (2017) [30]. In contrast, Mickle et al. (2008) [27] observed flatter feet and greater plantar arch index values in overweight children aged 3 to 5 years.

The inconsistency across studies may be related to differences in assessing adiposity, as BMI alone does not capture body fat distribution. There is still a lack of evidence concerning how abdominal adiposity, in particular, influences foot posture in school-aged children. Considering that excess abdominal fat alters posture and load distribution, understanding its biomechanical impact is essential to prevent musculoskeletal disorders and reduce healthcare costs in the SUS [12,13]. Thus, this study aimed to investigate the relationship between abdominal adiposity and foot support loading among obese, overweight, and eutrophic school-aged children.

## 2. Methods

### 2.1. Sample and Study Design

A prospective cohort study was performed with 65 school children, aged 6–9 years, from a public school, southern region of Santo Amaro, São Paulo/SP. The participants were divided in groups according to body mass index (BMI) categories: eutrophic schoolchildren group (GE, n = 20; BMI ≥ 18.5 and <25.0); overweight schoolchildren group (GOW, n = 20; BMI ≥ 26.0 and <29.0); and obese schoolchildren group (GO, n = 25; BMI ≥ 30.0).

The inclusion criteria were children enrolled in public school from grades 1 to 5; aged between 6 and 10 years; agreement to the study conditions; and cognitive ability to understand the instructions provided. The exclusion criteria were symptomatic lower-limb musculoskeletal conditions, central or peripheral nervous system disorders, diabetes mellitus, rigid foot deformities, foot or ankle injections in the past three months, previous or upcoming surgery within twelve months, allergy to insole materials, or intellectual disability.

This study was approved by the Ethics Committee on Human Research of the Universidade Santo Amaro (UNISA), under approval number 4.350.739. All participating children had the consent of their parents or legal guardians, who signed the informed consent form prior to participation, in accordance with Resolution 466/12 of the National Health Council. The assessments were conducted at the Biomechanics and Musculoskeletal Rehabilitation Laboratory at Universidade Santo Amaro (UNISA).

### 2.2. Clinical Evaluation Protocol

First, when the child arrived at the data collection site, and after the parents and/or guardians signed the consent form, initial contact was made with the children, accompanied by the school’s pedagogical director, for an initial interview and characterization of anthropometric measures and any possible orthopedic injuries. This was carried out through a questionnaire and a physical assessment of body weight. Subsequently, an appointment was scheduled for a clinical evaluation with the doctor and an abdominal ultrasound examination.

#### 2.2.1. Foot Posture Index-FPI Evaluation Protocol

The assessment of foot posture was conducted using the Foot Posture Index (FPI), a clinical diagnostic tool designed to quantify the degree to which a foot can be classified as supinated, pronated, or neutral [29]. The child was in an orthostatic position with bipodal support, and a 7.5 cm EVA foam rectangle was placed between the feet to standardize the plantar support surface. The child was instructed to align their upper limbs on the trunk. Adherence to posture was emphasized and any movement or body inclination can significantly alter the results.

The evaluations were performed by a single trained physiotherapist, who assigned values based on a series of observations made in three regions of the foot: the hindfoot, midfoot, and forefoot. Positive values (+2) indicate a pronated foot posture, negative values (−2) indicate a supinated foot posture, and a score of zero indicates a neutral foot posture. Each of the six criteria was graded on a scale of 0 (neutral), +1 or +2 (pronated), and −1 or −2 (supinated). The result for each criterion contributes to the overall foot posture index. A high positive score indicates a pronated foot (+6 to +9, highly pronated +10+), a high negative score indicates a supinated foot (−1 to −4, highly supinated −5 to −12), and a neutral score is close to zero (0 to +5). The final score ranges from −12 to +12, with each criterion being assessed independently [29,30]. Intra-rater reliability was tested with a 7-day interval and was classified as substantial (Kappa = 0.80). Inter-rater reliability was tested on different days owing to the logistics of the research and was classified as moderate (Kappa = 0.78).

**The Criteria Evaluated Were as Follows [29,30]:**

**(A)**  **Palpation of the Talus Head**

The talus head is palpated both medially and laterally at the anterior level of the ankle. To palpate the medial region, an imaginary line is drawn between the navicular tubercle and the medial malleolus. For the lateral region, the anterior border of the lateral malleolus is identified, and the palpation is extended slightly forward and medially.

**(B)**  **Supra and Inframalleolar Lateral Curvature**

The curves above and below the lateral malleolus in the posterior region of the ankle are observed. In a neutral foot, these curves should be approximately similar. In a pronated foot, for example, the lower curve is more pronounced than the upper one due to foot abduction and calcaneal eversion. In a supinated foot, the opposite is observed. A ruler can be used to estimate the malleolar curve. In cases of edema or obesity, this curve may disappear, and it should then be evaluated as zero or not considered in the final.

**(C)**  **Position of the Heel in the Frontal Plane**

In the posterior region, the position of the calcaneus is observed. The Achilles tendon serves as a reference. Any material that represents a straight line perpendicular to the axis of the foot can also be used.

**(D)**  **Prominence of the talonavicular joint**

The region of the talonavicular joint is located. In a normal foot, this area is flat; in a pronated foot, it is convex; and in a supinated foot, it is concave.

**(E)**  **Height and congruence of the medial longitudinal arch**

The main element of this observation is the congruence of the arch and, secondarily, its height. In the neutral foot, this curvature is relatively uniform. If the foot is supinated, the curve becomes sharper at the posterior level, and when pronated, this arch flattens at the central level and the metatarsal joints.

**(F)**  **Abduction and adduction of the forefoot in relation to the hindfoot**

When the foot is viewed posteriorly in line with the longitudinal axis of the calcaneus in the neutral foot, the same portion of the forefoot is observed at the medial and lateral levels. In the supinated foot, the forefoot is adducted, allowing greater visibility of the medial region, while the opposite occurs in the pronated foot. It is important that the examiner positions themself in the center of the calcaneus to continue the observation and, if there is a fixed adduction of the forefoot, caution should be taken and the metatarsophalangeal joints observed.

#### 2.2.2. Ultrasound of Subcutaneous and Intra-Abdominal Fat

**Figure 1:**
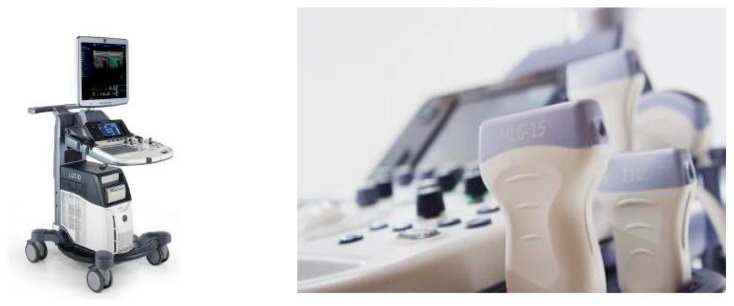
Logią S7 Expert model device, from General Electric Company and linear ML6-15 MHZ and convex C (1.5 MHz) transducers.

All the children were subjected to the scheduled evaluation and ultrasound examination. The children were examined in the supine position, without the need for fasting, using a Logiq S7 Expert ultrasound machine from the General Electric Company (GE) (Boston, MA, USA), with a linear ML transducer (6–15 MHz) and a convex C transducer (1–5 MHz) (Figure 1), following the methodology described by Sakuno et al. (2014) [32].

The ultrasound device was adjusted using a time gain compensation curve in the neutral position, and overall gain was calibrated so that liquid structures, such as the gallbladder, inferior vena cava, and aorta, appeared anechoic. All measurements were performed without pressure on the transducer and recorded in centimeters (cm). Intra-rater reliability was tested (ICC = 0.80). Inter-rater reliability was tested on different days and classified as higher (ICC = 0.88). Each measurement was determined as the arithmetic mean of three readings [32]:

**Subcutaneous cellular tissue (SCCT):** Distance from the skin to the linea alba, measured along the hemisternal line, 1 cm above the umbilical scar, using a linear transducer in the longitudinal plane.

**Minimal SCCT:** Thickness at the thinnest point of the SCCT along the hemisternal line above the umbilical scar, measured with a linear transducer in the longitudinal plane.

**Maximum SCCT:** Thickness at the thickest point of the subcutaneous tissue along the hemisternal line above the umbilical scar, measured with a linear transducer in the longitudinal plane.

**Preperitoneal fat (PPF):** Distance from the linea alba to the anterior parietal peritoneum along the hemisternal line, 1 cm above the umbilical scar, using a linear transducer in the longitudinal plane.

**Minimum PPF:** Thickness at the thinnest point between the linea alba and anterior parietal peritoneum along the hemisternal line above the umbilical scar, measured with a linear transducer in the longitudinal plane.

**Maximum PPF:** Thickness at the thickest point between the linea alba and anterior parietal peritoneum along the hemisternal line above the umbilical scar, measured with a linear transducer in the longitudinal plane.

**Intraperitoneal fat (IPF):** Measured with a convex transducer in three ways:

**IGap:** Distance between the anterior peritoneum and anterior wall of the aorta along the hemisternal line, 1 cm above the umbilical scar, in cross-section.

**IGp:** Distance between the anterior peritoneum and posterior wall of the aorta along the hemisternal line, 1 cm above the umbilical scar, in cross-section.

**IGpo:** Thickness of the lesser omentum, measured as the distance between the posterior surface of the left liver lobe and the anterior wall of the aorta at the level of the celiac trunk in the epigastric midline, in longitudinal section.

**Figure 2:**
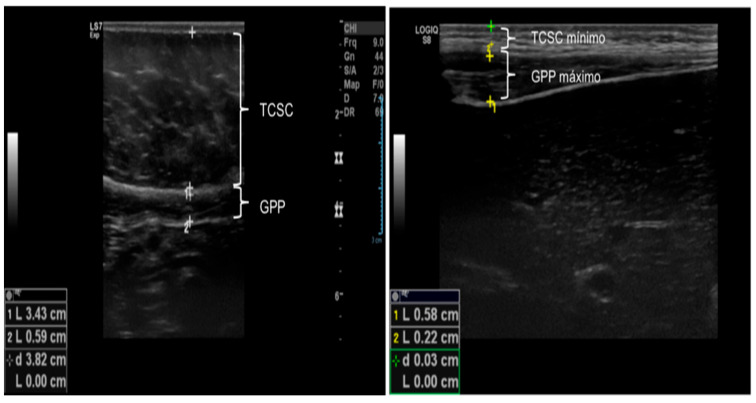
Illustration of ultrasound measurements: SCCT: Subcutaneous Cellular Tissue; PPF: Preperitoneal Fat.

**Figure 3:**
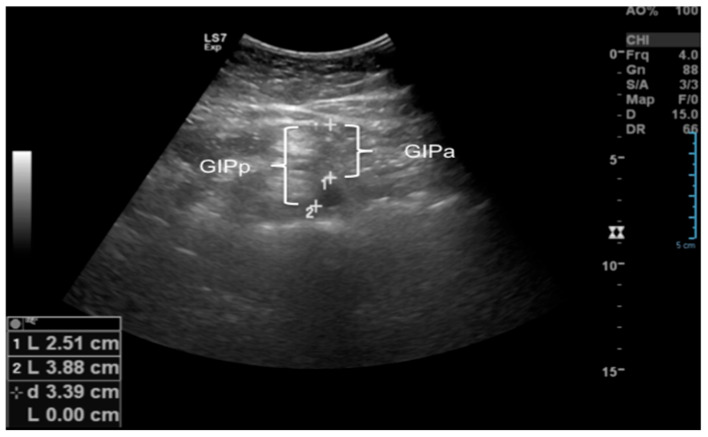
Illustration of ultrasound measurements: IGap: Intraperitoneal fat in relation to the anterior wall of the Aorta artery. IGp: Intraperitoneal fat in relation to the posterior wall of the Aorta artery.

**The abdominal wall fat index (ABFI)** was calculated as the ratio of the largest PPF measurement to the smallest SCCT measurement (Figure 2 and Figure 3).

## 3. Statistical Analysis

All statistical analyses were conducted using SPSS version 24 (IBM, Chicago, IL, USA). Normality of the data was assessed using the Shapiro–Wilk test. The sample size calculation was based on the Foot Posture Index, assuming a moderate effect size (F = 0.25), 80% power, and a 5% significance level. To compare dependent variables between groups, a one-way analysis of variance (ANOVA) for independent measures was performed, followed by Tukey’s post hoc test. Simple linear regression analyses were conducted to evaluate the association between abdominal adiposity and the Foot Posture Index. Inter- and intra-rater reliability were assessed using the Intraclass Correlation Coefficient (ICC) and Cohen’s Kappa. A *p*-value < 0.05 was considered statistically significant for all analyses.

## 4. Results

Anthropometric variables related to age and height did not differ significantly between obesity, overweight, and eutrophic groups. Significant differences were observed only for body mass and body mass index, with the obesity and overweight groups differing from the eutrophic group, but no differences between the obesity and overweight groups (Table 1).

In Table 2, a significant difference in the feet (right and left) was observed between the groups: GE, GOW, and GO. The obese and overweight children demonstrated a pronated foot posture, that is, with more medial support in relation to the eutrophic children, who presented neutral feet (*p* > 0.001). More medial support of the feet leaves the child with a greater impact of the forces on the ankle–foot and knee joints, especially in static and dynamic postures.

Table 3 presents significant differences between the abdominal adiposity variables (TCSC, TCSC min., TCSC max., PPF max., IGap, IGp, and IGpo) among the groups: GE, GOW, and GO, except for the PPF min. and PPF max. variables. Obese and overweight children demonstrated increased abdominal fat thickness compared to eutrophic children.

**Figure 4:**
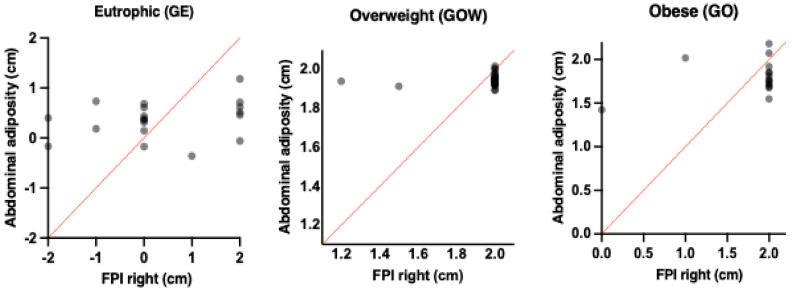
Demonstration of associations between Abdominal adiposity and FPI right between the different groups: Eutrophic (GE), Overweight (GOW) and Obese (GO).

Table 4 observed a moderately significant correlation between the abdominal adiposity thickness in obese children (OG) and the support of the more pronated right foot. In addition, a slight but significant correlation can be observed between the abdominal adiposity of overweight children and the support of the more pronated right foot, as shown in Figure 4. Eutrophic children did not show a positive correlation with the support posture of the feet.

**Figure 5:**
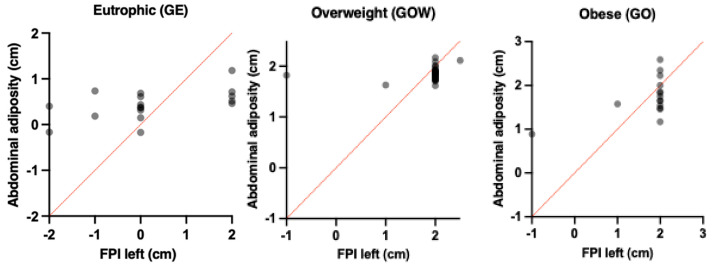
Demonstration of associations between Abdominal adiposity and FPI left between the different groups: Eutrophic (GE), Overweight (GOW) and Obese (GO).

Table 5 shows a moderate and significant correlation between the abdominal adiposity thickness in obese children (OG) and the support of the more pronated left feet. In addition, a good and significant correlation can be observed between the abdominal adiposity of obese children and the support of the more pronated left feet, as shown in Figure 5. Eutrophic children did not show a positive correlation with the support posture of the feet.

## 5. Discussion

This study aimed to evaluate the impact of abdominal adiposity and its association with the foot support posture of obese, overweight, and eutrophic schoolchildren. The main findings showed that the thickness of abdominal adiposity promoted a more pronated foot posture in obese and overweight children compared to eutrophic children, who presented neutral feet. Another important finding was that increased abdominal adiposity predicted a more pronated foot posture, both in the right and left feet, making children more vulnerable to the development of orthopedic dysfunctions in the ankle–feet and knee segments, especially inflammatory issues due to overload forces and ligamentous tension in the ankle ligaments.

These findings may be explained by several biomechanical mechanisms. Increased abdominal adiposity may shift the body’s center of mass anteriorly, resulting in compensatory changes in lower-limb alignment and foot posture [31]. Excess body mass may also increase ligamentous laxity and reduce proprioceptive control, leading to greater pronation and medial loading of the feet. This interpretation is supported by recent pediatric biomechanical studies showing that foot type and postural control in children are influenced by body composition, ligament laxity, and muscle strength [33,34].

Overweight and obesity in children and adolescents are growing public health concerns, with an increasing number of patients presenting comorbidities, as reported by pediatricians and orthopedic surgeons. According to evidence from the literature, flat or pronated feet and persistent valgus knees are more common in overweight children, as is spinal pain and osteoporosis in overweight and obese children [17,35]. Our results align with previous findings that excess adipose tissue alters plantar pressure distribution, especially in the medial midfoot region [36,37]. These dysfunctions may worsen during puberty, particularly in adolescence, which underscores the importance of the current study by showing that obese and overweight children, with increased abdominal adiposity, are more likely to present pronated foot support, making them more susceptible to orthopedic dysfunctions and pain in the feet and knees.

Another key finding in this study was that obese children had greater abdominal adiposity compared to overweight and eutrophic children. This finding is particularly relevant, as increased abdominal adiposity not only increases impact forces on the lower limb joints but may also displace the center of gravity, destabilizing the vectors of forces received by the foot support, especially during static postures and walking. Evidence of the association between overload on the lower extremities and gait patterns has already been established in obese adults, with the medial region of the feet being most affected and receiving greater force vectors compared to eutrophic adults [38,39]. The current study extends this evidence to a pediatric population, confirming that even in school-age children, abdominal adiposity correlates with medialized load and pronated posture.

The current study, however, did not assess adults but rather school-age children, and the results corroborate the imbalance of impact forces received by the more pronated foot posture, which can be explained by the force vectors remaining more medial, thereby overloading the midfoot region and the medial side of the knee joint.

According to the literature, overweight children and adolescents, during the growth of the bone, may experience alterations in foot support patterns and plantar load distribution [36,37], a pattern further supported by recent analyses of foot morphology in children showing that body weight influences arch height, plantar contact area, and gait symmetry [31,33,34]. It is therefore evident that the morphology of the foot in obese children is characterized by a reduction in the longitudinal arch, resulting in flat (pronated) feet, especially during walking [37,38,39]. In static postures, obese children show greater foot load compared to non-obese children and foot and ankle pathology. [40,41,42,43]. The findings suggest that excess weight increases stress on soft tissues and joints, leading to greater foot discomfort and pain, and potentially limiting physical activity [17,44,45].

According to Butterworth et al. (2014) [46], increased fat mass is significantly associated with foot pain and dysfunction, increasing the vulnerability to musculoskeletal disorders in this segment. Based on the scientific evidence mentioned above, the first understanding of foot support comes from the foot posture characteristics (pronated, supinated, and neutral). To date, no studies have been found in the literature correlating increased abdominal thickness, quantified by ultrasound, with foot support in obese and overweight children.

In the findings of this study, a positive cause-and-effect relationship was observed, i.e., the increase in abdominal thickness in obese and overweight children leads to an increase in pronation of both the right and left feet. According to Catan et al. (2020) [47], overweight and obese children and adolescents experience an increase in impact force on the midfoot region, decreasing sensitivity across the entire foot, leading to changes in foot function.

Clinically, these findings underscore the importance of early screening for foot posture abnormalities in overweight and obese children. Targeted interventions—such as neuromuscular and proprioceptive training, strengthening of intrinsic foot muscles, and orthotic management—may help reduce excessive pronation and its downstream consequences on the knee and hip joints. Our findings align with the recommendations of Molina-García et al. (2022) [48], who emphasized that integrative neuromuscular training could correct altered plantar support patterns in children with excess weight. Incorporating these preventive strategies into pediatric rehabilitation and school-based physical activity programs could help mitigate long-term orthopedic risks. Early identification of children with excess abdominal fat may enable clinicians and educators to implement targeted interventions aimed at improving postural alignment, balance, and lower-limb function. Preventive measures such as tailored physical exercise programs, postural education, weight management initiatives, and regular physical activity could reduce the risk of musculoskeletal overload and postural deviations during growth. Furthermore, rehabilitation programs focusing on strengthening, balance, gait training, and lower-extremity flexibility may help minimize potential biomechanical compensations and improve overall functional outcomes.

This study presents some limitations that should be acknowledged. First, the absence of dynamic and functional assessments—such as gait analysis on plantar pressure mapping—limits the understanding of how abdominal adiposity and foot pronation interact during movement. The cross-sectional design also restricts causal inference, performed in a public reference school, preventing conclusions about the temporal sequence between increased adiposity and postural adaptations. Thus, the relatively small sample size may reduce the generalizability of the findings. These methodological constraints are consistent with limitations reported in recent pediatric biomechanical studies [17,38,39,40,41], which emphasize the importance of longitudinal and multi-modal approaches to better elucidate these complex interactions. Future research should therefore include dynamic gait assessments, motion capture analysis measurements and shoe control, with longitudinal study designs, to determine causal pathways and evaluate the effectiveness of targeted interventions.

## 6. Conclusions

Greater abdominal adiposity was associated with a more pronated foot posture in obese and overweight schoolchildren. This finding suggests that central obesity in childhood may alter lower-limb biomechanics and increase medial plantar loading, predisposing to pain and orthopedic dysfunctions in the foot and ankle segments. Early pediatric screening and targeted interventions—such as postural education and the implementation of corrective exercise programs—may help mitigate these biomechanical alterations and prevent future musculoskeletal complications.

## Figures and Tables

**Table 1:** Mean, standard deviation, and comparisons between the different groups of obesity and overweight in relation to the eutrophic group for the anthropometric characteristics of schoolchildren.

Variables	GE (n = 20)	GOW (n = 20)	GO (n = 25)	*p*
Age (years)	13.4 ± 1.3	14.2 ± 1.3	14.2 ± 1.3	0.448
Weight (kg/cm^2^)	46.0 ± 8.6	50.1 ± 7.4	50.1 ± 7.4	0.019 *
Height (cm)	1.5 ± 0.7	1.6 ± 0.7	1.6 ± 0.7	0.123
BMI (kg/m^2^)	18.2 ± 3.6	19.6 ± 2.3	19.6 ± 2.3	0.001 *

* One-way ANOVA test, considering statistical differences between GE versus GOW and GE versus GO, *p* < 0.05.

**Table 2:** Mean, standard deviation and comparisons of the eutrophic (GE), overweight (GOW), and obese (GO) groups for the foot support index of schoolchildren.

Foot	GE (n = 20)	GOW (n = 20)	GO (n = 25)	*p*
FPI right	0.4 ± 0.2	1.8 ± 0.6	1.9 ± 0.3	0.001 *
FPI left	0.5 ± 0.2	1.7 ± 0.8	2.0 ± 0.8	0.024 *

* One-way ANOVA test, considering statistical differences *p* < 0.05.

**Table 3:** Mean, standard deviation and comparisons of the eutrophic (GE), overweight (GOW), and obese (GO) groups for abdominal adiposity of schoolchildren.

Abdominal Ultrasound	GE (n = 20)	GOW (n = 20)	GO (n = 25)	*p*
Subcutaneous cellular tissue (SCCT, cm)	0.43 ± 0.28	1.03 ± 0.71	1.66 ± 0.75	0.001 *
Minimum subcutaneous cellular tissue (minimum SCCT, cm) SCCT	0.34 ± 0.16	0.65 ± 0.48	1.08 ± 0.51	<0.001 *^&^
Maximum subcutaneous cellular tissue (maximum SCCT, cm)	0.56 ± 0.34	1.19 ± 0.77	1.83 ± 0.78	0.010 *
Preperitoneal fat (PPF, cm)	0.12 ± 0.04	0.14 ± 0.09	0.15 ± 0.09	0.583
Minimum preperitoneal fat (minimum PPF, cm)	0.12 ± 0.03	0.12 ± 0.07	0.12 ± 0.05	0.101
Maximum preperitoneal fat (maximum PPF, cm)	0.58 ± 0.27	0.76 ± 0.25	0.91 ± 0.26	<0.001 *
Intra-peritoneal fat (IPFa—anterior wall of the aorta, cm)	3.41 ± 0.66	3.66 ± 0.96	4.41 ± 1.17	0.003 *^&^
Intraperitoneal fat (IPFp—posterior wall of aorta, cm)	4.21 ± 0.73	4.50 ± 1.17	5.33 ± 1.22	0.028 *
Intraperitoneal fat (IPF—lesser omentum, cm)	0.81 ± 0.15	0.94 ± 0.24	1.15 ± 0.33	0.001 *^&^
Total abdominal fat (cm)	4.76 ± 0.80	5.65 ± 1.55	7.13 ± 1.64	<0.001 *^&^

* One-way ANOVA test, considering statistical differences *p* < 0.05.

**Table 4:** Simple linear regression to verify the relationship between abdominal adiposity of each group (obesity—GO; overweight—GOW; and eutrophic—GE) and the support index of the right foot of schoolchildren.

Groups	Abdominal Adiposity (cm)	FPI Right (cm)	β	t	r	r^2^	IC	*p*
Eutrophic (GE)	4.76 ± 0.80	0.4 ± 0.2	2.5	1.0	0.04	0.00	0.2;1.0	0.563
Overweight (GOW)	5.65 ± 1.55	1.8 ± 0.6	0.15	0.3	0.12	0.01	1.8;2.0	0.018 *
Obese (GO)	7.13 ± 1.64	1.9 ± 0.3	0.11	2.0	0.41	0.16	1.4;2.1	0.005 *

* Simple linear regression test, considering significant differences *p* < 0.05.

**Table 5:** Simple linear regression to verify the relationship between abdominal adiposity of each group (obesity—GO; overweight—GOW; and eutrophic—GE) and the support index of the left feet of schoolchildren.

Groups	Abdominal Adiposity (cm)	FPI Left (cm)	β	t	r	r^2^	IC	*p*
Eutrophic (GE)	4.76 ± 0.80	0.5 ± 0.2	1.8	0.49	0.06	0.00	0.2;1.1	0.630
Overweight (GOW)	5.65 ± 1.55	1.7 ± 0.8	0.15	1.6	0.21	0.04	1.6;2.2	0.029 *
Obese (GO)	7.13 ± 1.64	2.0 ± 0.8	0.35	1.9	0.54	0.29	1.2;2.1	0.034 *

* Simple linear regression test, considering significant differences *p* < 0.05.

## Data Availability

Data is available upon request addressed to the contact author.
